# Associations between depression, anxiety, stress, hopelessness, subjective well-being, coping styles and suicide in Chinese university students

**DOI:** 10.1371/journal.pone.0217372

**Published:** 2019-07-01

**Authors:** Bob Lew, Jenny Huen, Pengpeng Yu, Lu Yuan, Dong-Fang Wang, Fan Ping, Mansor Abu Talib, David Lester, Cun-Xian Jia

**Affiliations:** 1 Department of Social Psychology, Faculty of Human Ecology, Putra University of Malaysia, Serdang, Selangor, Malaysia; 2 Department of Social Work and Social Administration, The University of Hong Kong, Hong Kong, China; 3 Department of Epidemiology, School of Public Health, Shandong University & Shandong University Center for Suicide Prevention Research, Jinan, Shandong, China; 4 Department of Preventive Medicine, School of Basic Medical Sciences, Shandong University of Traditional Chinese Medicine, Jinan Changqing, Shandong, China; 5 Information Network Center, Qilu Hospital of Shandong University, Jinan, China; 6 Department of Human Development and Family Study, Putra University of Malaysia, Serdang, Selangor, Malaysia; 7 Stockton University, Galloway, New Jersey, United States of America; Universita degli Studi Europea di Roma, ITALY

## Abstract

Suicide is a major public health concern worldwide. This study aimed to predict the suicidal behavior of Chinese university students by studying psychological measures such as hopelessness, orientation to happiness, meaning in life, depression, anxiety, stress, and coping styles. In November 2016, a stratified-clustered-random sampling approach was utilized to select subjects from two large public medical-related universities in Shandong province, China. This sample consisted of 2,074 undergraduate students (706 males, 1,368 females; mean age = 19.79±1.39 years). The students’ major risk factors for suicide were depression, anxiety, stress, and hopelessness, and the students’ minor risk factors included orientation to happiness and coping styles (including self-distraction, self-blame and substance use). Notably, the presence of meaning in life had a positive effect on preventing suicide and acted as a protective factor, which suggests that it is important to identify risk factors as well as protective factors relevant to the target population group in order to increase the effectiveness of counseling and suicide prevention programs.

## Introduction

Globally, suicide has been recognized as a severe public health concern associated with significant disability, psychosocial impairment, and medical illness. Approximately 800,000 people take their own lives each year [1]. Furthermore, suicide rates among adolescents and young adults, especially on university campuses, have been increasing at an alarming rate [2]. Significant precursors to dying by suicide are nonlethal suicidal behaviors—such as suicide ideation and suicide attempts [3–5]. A report from the USA in 2014 claimed that an estimated 108,000 full-time college students had attempted suicide [6]. Statistics show the prevalence of suicide ideation or suicide plans among college students to range between 5.4–38% worldwide [7–11]. In China, suicide is the leading cause of death for young adults [12]. The suicide rate in China has been decreasing but remains the second leading cause of death for this age group [13].

University students constitute a unique group due to their relatively high intelligence, aspirations and self-esteem. However, they are in a developmental period, transitioning from adolescence to adulthood, and they must adapt to new living conditions as well as new social and academic challenges [14]. Many of them develop mental disorders, some of which are serious [15, 16]. When confronted with adverse life events, a tense campus climate and psychological stress can exacerbate the stress response, and extreme reactions such as suicidal behaviors are likely to occur [17].

Previous studies have revealed that an increased risk of suicidal behavior in university students is correlated with several identifiable factors, such as personality traits, substance abuse, negative life events (such as academic difficulties) [18], social disconnectedness (poor interpersonal networks) [19], conflict in their families [20, 21] and medical illness, which can include mental disorders or physical disorders (such as a stroke) [22]. In a systematic review investigating the relationship between suicide and stroke, stroke as a medical disorder was identified as a significant risk factor for suicidality (including both suicidal ideation and suicidal behavior), especially among depressed patients [22]. The existence of previous mood disorders, a prior history of stroke, and cognitive impairment were found to be important risk factors for suicide [22]. In other studies with undergraduate students, a population group that normally has fewer physical health problems, variables such as hopelessness [23, 24], anxiety [25], and depression [6, 26] appear to be the more important risk factors. Subjective well-being factors, such as those related to meaning in life [27] and limited coping abilities [28, 29], are also potential predictors of suicidality.

The present study was designed to explore the association between a number of psychological factors and suicidal behaviors among university students in China, which could inform prevention strategies by better understanding the key risk factors and protective factors that are relevant to university students. Specifically, the main study purpose was to examine whether risk factors including hopelessness, depression, anxiety, stress, coping styles, and protective factors (including orientation to happiness, meaning in life) were significantly associated with suicidal thoughts and behaviors in our sample group.

## Methods and material

### Subjects and procedure

This study was conducted in November 2016 in Jinan, the capital city of Shandong province, China. Shandong province lies to the south of Beijing and has approximately 100 million residents, of whom there are approximately 7.23 million people living in Jinan [30]. Two large public medical-related universities in Jinan were selected, and two large faculty colleges from each university were sampled for this study. A stratified-clustered-random sampling approach was then undertaken to choose participants from three or four classes in each grade (i.e., freshman, sophomore, junior and senior levels). All students in the sampled classes were invited to complete the self-report questionnaires. It took each respondent approximately 15 minutes to complete the questionnaire during class breaks. The study was approved by the institutional review boards and by the Ethics Committee at Shandong University School of Public Health.

Of the 2,197 questionnaires distributed, 2,074 responses were received from undergraduate students, resulting in a response rate of 94.4%. Among the respondents, 706 were males (mean age = 19.86, SD = 1.50 years) and 1,368 were females (mean age = 19.75, SD = 1.33 years). In China, females are the dominant participants in higher education, and it is therefore common for the proportion of female students in universities to exceed that of male students. The mean age of all respondents was 19.79 (SD = 1.39 years). The sample consisted of 574 (27.7%) freshmen, 521 (25.1%) sophomores, 619 (29.8%) juniors, and 360 (17.4%) seniors. Regarding ethnicity demographics, 1,939 (93.5%) were Han Chinese and 135 (6.5%) were from ethnic minorities.

### Measures

The Suicidal Behaviors Questionnaire-Revised (SBQ-R) comprises four items, each assessing a different suicidal behavior: lifetime suicide ideation and suicide attempts; frequency of suicide ideation over the past twelve months; threats of suicide attempt; and likelihood of suicidal behavior in the future [31]. The total score, ranging from 3 to 18, could be used to evaluate the suicide risk for undergraduate students [32]. The Chinese version of the SBQ-R was translated by our research team. In this study, the Cronbach alpha value of the SBQ-R was 0.67.

The Beck Hopelessness Scale (BHS) is a 20-item instrument measuring one’s negative attitudes about the future [33]. It was modified from yes/no to a 5-point Likert scale ranging from 1 (strongly agree) to 5 (strongly disagree) in a study with Chinese respondents [34]. The Chinese version of the BHS had satisfactory reliability and validity in adolescents [35], similar to the findings of this study (α = 0.90) based on the 5-point Likert scale.

The Orientation to Happiness Questionnaire (OTH) has 12 items that evaluate two different strategies for pursuing happiness (6 items per strategy): a life of pleasure (e.g., “In choosing what to do, I always take into account whether it will be pleasurable.”) and a life of meaning (e.g., “My life serves a higher purpose.”) [36]. Using the Chinese version of the OTH, responses were given on a 5-point Likert scale ranging from 1 (very much unlike me) through 5 (very much like me), and higher subscale scores demonstrate a stronger endorsement of the corresponding orientation [37]. The Cronbach alpha value of the OTH was 0.84.

Meaning in life was measured by the Chinese version of the Meaning in Life Questionnaire (MLQ) [38]. It is made up of two five-item subscales [39]: the presence of meaning in life (MLQ-P) and a search for meaning in life (MLQ-S). All ten items are rated from 1 (absolutely untrue) to 7 (absolutely true). The item scores are then summed, and higher scores indicate a stronger correspondence to meaning in life [40]. In this study, the Cronbach alpha value of the MLQ was 0.88 (0.78 and 0.88 for the MLQ-P and MLQ-S respectively), showing satisfactory internal consistency.

The Depression Anxiety Stress Scales (DASS) [41] is a 42-item questionnaire including three subscales to measure three different negative emotional states, namely, depression, anxiety and stress. A revised version of the DASS, the DASS-21, was used in this study; it has seven items for each subscale and has been widely used for the identification and assessment of the corresponding emotional symptoms [42–44]. The score range for each item varies from 0 (did not apply to me at all) to 3 (applied to me very much or most of the time). A lower score reflects better mental health. Studies have shown the DASS-21 to be a reliable and valid inventory and suitable for studies in Chinese college students [45]. In this study, the Cronbach alpha values of the DASS were 0.84, 0.82, and 0.79 for the depression, stress and anxiety subscales, respectively.

The Brief COPE Scale (BCS) is a multidimensional scale [46], presenting 14 subscales, each with two items measuring a strategy that one would take under some certain kind of stressor. The fourteen coping strategies included in the Brief COPE are self-distraction, active coping, denial, use of instrumental support, substance use, use of emotional support, positive reframing, behavioral disengagement, venting, planning, humor, acceptance, religion, and self-blame. It has been translated into various languages and used in different countries [47, 48]. In this study, an eleven-factor model [47] was adopted to measure situational and dispositional coping responses.

### Statistical analysis

Statistical analyses were performed using IBM SPSS Statistics for Windows, Version 24.0 (IBM corp., and Armonk, New York, US). Cronbach’s alpha was used to measure the reliability of scales.

Multiple linear regression with stepwise selection method was used to explore significant predictors of suicidal behaviors. Age, gender, and all psychological variables, including hopelessness, depression, anxiety, stress, coping styles, orientation to happiness, and meaning in life were simultaneously entered into the multiple regression model to predict suicidal behaviors with those weak predictors being removed stepwise to result in a final model containing all significant predictors. Furthermore, the variance inflation factor (VIF) and tolerance were calculated to determine the presence of multicollinearity.

All relevant data are in Supporting Information.

## Results

The mean scores and standard deviations for each of the scales are shown in Table 1, along with Cronbach’s alphas for each scale. The high Cronbach’s alphas indicated satisfactory reliability for the instruments.

**Table 1:** Mean scores, standard deviations and Cronbach alpha reliabilities on suicidal behaviors, depression, anxiety, stress, hopelessness, subjective well-being and coping scales.

Variables	Mean	SD	α
Suicidal behaviors	4.86	2.47	0.67
Hopelessness	41.77	11.96	0.90
Orientation to happiness			
Life of meaning	20.74	4.40	0.81
Life of pleasure	20.14	4.46	0.80
Meaning in life			
Presence of meaning	26.19	5.57	0.78
Search for meaning	27.09	5.79	0.88
Depression	3.16	3.56	0.84
Stress	5.36	4.08	0.82
Anxiety	4.75	3.77	0.79
Coping styles			
Problem-solving	12.89	2.39	0.77
Accommodation	12.34	2.44	0.75
Support-seeking	11.50	2.72	0.80
Behavioral disengagement	3.65	1.39	0.63
Denial	3.68	1.38	0.51
Self-distraction	5.78	1.44	0.53
Self-blame	5.05	1.43	0.55
Humor	4.15	1.50	0.49
Venting	5.31	1.49	0.49
Substance use	2.79	1.38	0.79
Religion	3.57	1.43	0.47

*Note*. SD: standard deviation

*α*: Cronbach’s alpha.

The scale scores were subjected to multiple regression, which shows the significant components for the prediction of SBQ. Collinearity diagnosis showed a satisfactory variance inflation factor (VIF<5) and tolerance in multiple regression. The strongest predictors of suicidal behavior were hopelessness, depression, stress and some coping styles like self-distraction and self-blame (see Table 2). As a protective factor, the presence of meaning in life showed the potential to prevent suicide. This was also consistent with the Pearson correlations, the highest of which were associated with hopelessness, depression and stress. Moreover, we found that age and gender were statistically significant for regression beta, but orientation to happiness had few contributions to suicidality.

**Table 2:** Correlations and regressions between suicide behavior questionnaire scores and depression, anxiety, stress, hopelessness, subjective well-being and coping styles scale scores.

	Pearson *r*	Regression beta	Multicollinearity test	
			Tolerance	VIF
Age	-0.04	-0.07 *	0.99	1.01
Gender	0.03	0.45 ***	0.96	1.05
Hopelessness	0.31 ***	0.03 ***	0.59	1.70
Presence of meaning	-0.22 ***	-0.03 **	0.75	1.33
Depression	0.38 ***	0.14 ***	0.34	2.95
Stress	0.34 ***	0.06 **	0.41	2.42
Coping styles				
Self-distraction	0.05 *	0.08 *	0.87	1.15
Self-blame	0.18 ***	0.14 ***	0.87	1.14
Multiple *R*: 0.43				

*Two-tailed *p* < 0.05

**two-tailed *p* < 0.01

***two-tailed *p* < 0.001

VIF: variance inflation factor.

## Discussion

The main findings of the present study were the following: the major risk factors for suicidal behavior were shown to be hopelessness, depression, stress and negative coping styles; furthermore, perceiving meaning in life was shown to be a protective factor for suicidal behavior.

The linear regression indicated that hopelessness, depression, stress and the presence of meaning in life were significant predictors of suicidal behavior. It has been well documented in suicidology literature that the greater the severity of depression and the higher the levels of hopelessness and stress, the greater is the likelihood of suicidal behavior in college students [26, 49–52]. In Furr’s research [53], approximately 53% of students stated that they had experienced depressive symptoms since entering into college, increasing the likelihood of suicidal ideation or behaviors. Lester [26] found that undergraduate students living on campus were more depressed than those living with parents or off campus. In the present study, the Depression subscale assessed dysphoria, devaluation of life, self-deprecation, lack of interest/involvement, anhedonia, and inertia. Consistent with most previous studies [52, 54], depression was a predictive factor for suicidal behavior among Chinese university students.

Hopelessness has been shown to be a risk factor for suicidal behavior, both nonlethal and lethal behavior types [23, 24, 55]. Individuals experiencing hopelessness have a pessimistic attitude about the future and react poorly to stressors, and hopelessness acts as a mediator between psychological distress and suicidal behaviors [56, 57].

Another well-documented predictor of suicidal behavior in university students is stress [26, 58]. University students face a barrage of stress through homework, projects and internships and thus have little time to join extracurricular activities for relaxation. They also must adjust to new living arrangements, many of which involve being away from home.

This present study found that having meaning in life was a protective factor for suicidal behavior, which was consistent with previous research [27, 59]. Having meaning in life may be defined as the pursuit and accomplishment of worthwhile goals and the accompanying satisfaction [60]. A sense of meaning contributes to the foundation of overall happiness and predicts positive traits and psychological strengths associated with fulfillment in life [61]. People without meaning in life tend to experience emptiness and have less motivation to continue to live [62, 63]. Thus, it is important to guide students to establish clear and reasonable life goals, to face and accept frustrations and setbacks in life, to release negative emotions and to optimize meaningful life situations [40] in order to lower the likelihood of future suicide.

Previous research has shown that negative coping styles were associated with suicidal behavior [29, 64]. Only some coping skills were found to have a weak relationship with suicidal behavior, and one reason for that finding may be poorly validated measures of coping skills. Furthermore, students with a high suicide risk tended to adopt passive coping skills when facing difficulties due to the lack of proper coping mechanisms to transfer their emotions.

The findings of the present study, based on the availability of useful measures to assess suicidal risk in university students in China, suggest that suicidal behaviors are significantly associated with several risk and protective factors. The data highlight the need to provide effective psychological outreach programs and suicide prevention measures and interventions for this population, taking into consideration the risk factors and protective factors of this age group. University students, young adults who have recently enrolled in institutions of higher education, might not yet have developed a mature perspective on life and death or have stable outlooks on suicide [27]. These programs and interventions should be conducted as soon as the students commence undergraduate programs and should be implemented on campus to reach more university students. In designing these preventive programs and interventions, it is important to screen students for risks and protective factors; to design good counseling techniques and content to address the multifacet sources that can increase risk factors; and to identify if an individual student possesses certain protective factor(s) so he or she can be placed in psychotherapy or counseling. For example, counseling and guidance for university students could draw on important protective factors, namely, meaning in life, to prevent suicidal behavior.

There are some limitations to this study. First, a cross-sectional study is insufficient to draw firm conclusions on causality, which is better studied using a longitudinal design that follows a cohort of individuals. Second, the samples were recruited from a limited region, and most of the selected subjects were medical students. Therefore, the results may not be representative of all university students. Third, there may have been potential biases associated with unmeasured confounding variables.

## Supporting information

S1 Data (XLSX)
